# Cucurbitacin B suppresses glioblastoma via the STAT3/ROS/endoplasmic reticulum stress pathway

**DOI:** 10.1038/s41598-025-21526-0

**Published:** 2025-10-27

**Authors:** Guangyao Lv, Shanshan Sun, Xueying Li, Ruxia Han, Shule Liu, Mei Lu, Xinfu Gao, Jianqiao Zhang, Wenwen Lv

**Affiliations:** 1 Department of Pharmacy, Binzhou Medical University Hospital, Binzhou, 256603 Shandong China; 2 School of Pharmacy, Binzhou Medical University, Yantai, 264003 Shandong China; 3 School of Health, Binzhou Polytechnic, Binzhou, 256600 Shandong China; 4 Clinical Trials Research Center, Department of Pharmacy, Binzhou Medical University Affiliated Zibo Central Hospital, Zibo, 255000 Shandong China

**Keywords:** Cucurbitacin b, Glioblastoma, Endoplasmic reticulum stress, Target therapy, Liposome, Cancer therapy, Tumour immunology, Cell death

## Abstract

Cucurbitacin B (CuB), the tetracyclic triterpenoid compound belonging to cucurbitaceae family, demonstrates significant anticancer properties across various tumor types. However, its efficacy against glioblastoma (GBM), particularly in overcoming blood-brain barrier (BBB) and unique tumor microenvironment, remains a critical area of investigation. Our study reveals that CuB causes GBM cell apoptosis via STAT3/ROS/endoplasmic reticulum (ER) stress pathway. To enhance targeted delivery, we developed M@CuB-Lips, a novel formulation of CuB encapsulated in cancer cell membrane-coated liposomes. In vitro and in vivo experimental results verified targeted M@CuB-Lips delivery to brain tumor sites, demonstrating significant tumor inhibition and enhanced effector T-cell infiltration. These findings underscore CuB’s potential in GBM therapy, particularly through STAT3 suppression and ROS-ER Stress pathway activation, while M@CuB-Lips offers a promising strategy for targeted drug delivery.

**Supplementary Information:**

The online version contains supplementary material available at 10.1038/s41598-025-21526-0.

## Introduction

Glioblastoma (GBM) is the most aggressive, recurring, and fatal subtype of grade IV malignant gliomas developing in the central nervous system^1^. Standard of care after diagnosis includes temozolomide (TMZ)-based concomitant chemotherapy, radiation, and maximum surgical resection^2^. However, TMZ still has significant negative effects including myelosuppression and tumor cells can evolve resistant to the medicine^3^. Consequently, there is an urgent clinical need for new therapeutic directions and medications for the treatment of GBM.

The signal transducer and activator of transcription 3 (STAT3) gene, a member of the STAT gene family, encodes a STAT protein that can be activated by a diverse array of cytokine receptors. Typically, STAT3 activation necessitates the phosphorylation of the Tyr705 residue. Persistent phosphorylation of STAT3 is observed in approximately 70% of human cancers, including GBM^4^. STAT3 plays a pivotal role in various processes such as epithelial-to-mesenchymal transition, proliferation, metastasis, cell cycle progression, stemness, and therapeutic resistance. Additionally, STAT3 activation leads to inadequate T-cell infiltration, which may contribute to the immunosuppressive microenvironment in GBM^5^. Consequently, inhibiting STAT3 activation represents a promising therapeutic approach for treating GBM.

Cucurbitacin B (CuB) is a traditional Chinese medicine monomer extracted from the cucurbitaceae plant^6^. It is also one of the most abundant cucurbitacins than other classes of cucurbitacin compounds, showing significant anti-tumor effects in a variety of malignant tumors. According to numerous studies, CuB’s anti-tumor effect is primarily achieved by preventing cell growth and proliferation, stopping the cell cycle, inducing cell apoptosis, disrupting the cytoskeleton, preventing cell migration and invasion, and inducing cell autophagy^7–9^. In recent years, multiple studies have demonstrated that CuB exerts anticancer effects in non-small cell lung cancer (NSCLC) and gastric cancer (GC) by targeting STAT3. For instance, Zeng et al. discovered that CuB induces ferroptosis in NSCLC cells by inhibiting STAT3 phosphorylation^10^, while Xu et al. reported that CuB impedes gastric cancer progression by directly suppressing STAT3 activity^11^. These findings suggest that CuB may play a role in the treatment of GBM through similar mechanisms.

The application of CuB in GBM treatment is limited by its poor water solubility and the barrier posed by the blood brain barrier (BBB)^12^. Liposomes, characterized by their low toxicity and immunogenicity, high biocompatibility, and biodegradability, serve as excellent carriers for drugs^13^. Furthermore, coating liposomes with tumor cell membranes can endow them with immune escape properties, thereby prolonging their circulation time in the bloodstream^14–16^. Additionally, due to the presence of proteins on their surfaces, tumor cell membranes inherit the homologous targeting function of the source cells, enabling them to be used for specific tumor-targeted therapy. Therefore, in this study, we prepared tumor cell membrane-coated CuB liposomes(M@CuB-Lips)for intracranial delivery in GBM.

## Materials and methods

### Material

Cucurbitacin B (purity ≥ 98%) was purchased from Chengdu Herbsubstance (China). CuB was dissolved in DMSO and stored at -20 °C for less than 1 month before use in vitro experiments. ROS Assay Kit, ATP Assay Kit were purchased from Beyotime (China).

## Cell source and culture

Human GBM cell lines U87MG were purchased from the cell bank of Chinese Academy of Sciences (China) and mouse glioma cell lines (GL261, Luc^+^GL261) were sourced from iCell Bioscience (China). Cells were grown in Dulbecco’s modified Eagle’s medium (Gibco, USA) supplemented with 10% fetal bovine serum (Procell, China), 100 U/mL penicillin, and 0.1 mg/mL streptomycin at 37 °C with 5% CO_2_. All cells were harvested in the exponential growth phase for assays.

## Growth suppression assays

For the cell viability assay, cells (4 × 10^3^ cells/well) were seeded into 96-well plates and treated with CuB at different concentrations. After 24–48 h treatment, cells were incubated with MTT and the absorbance was determined at 570 nm using microplate reader (BioTek, USA) and the values of IC_50_ were calculated by GraphPad software. For growth curves, cells (1 × 10^4^ cells/well) were plated in 6-well plates, treated with CuB at different concentrations, and counted over 6 d. Data was processed by GraphPad software.For colony formation assay, cells (1 × 10^3^ cells/well) were seeded in 6-well plates and treated with CuB at different concentrations for 24 h, after which the medium was replaced and the cells were allowed to grow for 10 d. The colonies were stained with Giemsa (Beyotime, China).

## Cell cycle assay

Cells (2 × 10^5^ cells/well) were plated in 6-well plates, treated with CuB at different concentrations for 24 h. After collected, cells refrigerated overnight in 70% ethanol. The cell cycle was determined by flow cytometry (FCM, BD Biosciences, USA) after cells were stained with PI.

## Western blotting assays

The cells, collected and lysed using RIPA lysis buffer containing 1% PMSF, were separated by centrifugation, and the resulting proteins were separated by SDS-PAGE and transferred to the PVDF membrane. After blocking, the membranes were incubated with primary antibodies included β-actin (GB15003, Servicebio, China), STAT3 (A1192, ABclonal, China), p-STAT3 (AP0705, ABclonal, China), eIF-2α (AF6771, Beyotime, China), p-eIF-2α (AF5803, Beyotime, China), CHOP (AF6684, Beyotime, China). The membranes were then incubated with the appropriate secondary antibody and the protein bands were detected using the ECL.

### ROS levels assays

The intracellular ROS levels were measured using a ROS Assay Kit. Briefly, cells (2 × 10^5^ cells/well) were seeded into 6-well plates to adhere overnight, followed by treatment with CuB for 24 h. The cells were incubated with DCFH-DA for 20 min at 37 °C and analyzed by fluorescence microscope or FCM.

## ATP levels assays

The supernatant ATP levels was detected by ATP bioluminescent assay kit. Briefly, cells (2 × 10^5^ cells/well) were seeded into 6-well plates to adhere overnight, followed by treatment with CuB for 24 h, the supernatant ATP levels were detected according to the manufacturer’s instructions.

## Cell apoptosis analysis

FCM analysis was performed using our previously published protocol^17^. Briefly, cells (2 × 10^5^ cells/well) were seeded into 6-well plates to adhere overnight, followed by treatment with CuB for 24 h. Cells were stained with PI/FITC-labeled annexin and analyzed by FCM.

### Antitumor activity of CuB in vivo

BALB/c-nu (female, 6–8 weeks old, 16–18 g) were purchased from Jinan pengyue laboratory animal breeding co.ltd (China). Animal care and experimental procedure in this study was conducted in accordance with the principles and procedures approved by the Animal Care and Use Committee of Binzhou Medical University Hospital (No.20240130-160) and were in compliant with ARRIVE guidelines. Mice were subcutaneously implanted into U87MG cells (2 × 10^6^ cells/per animal) to establish tumor xenograft models. After the tumor volume reached approximately 100 mm³, the mice were randomly divided into three groups: the control group received saline injections, the CuB group received daily intraperitoneal injections of CuB (0.5 mg/kg), and the positive control group received oral administration of TMZ (50 mg/kg) every two days for a total of seven doses. The weight and tumors volume of mice were measured every three days. After 25 days of treatment, the tumor-bearing nude mice were then humanely euthanized by isofurane followed by cervical dislocation by cervical dislocation. Finally, the tumors were removed from the muscles and dermis around them, weighed, and examined.

### Fabrication and characterization of M@CuB-Lips

CuB-Lip were produced through a straightforward film dispersion technique. For the studies of cell uptake and bio-distribution in vitro, Cy5.5-loaded Lips liposomes were prepared with the same procedure as above with the Cy5.5 concentration of 10 µg/mL. The isolation of membranes from GL261 cells followed the previously established protocol^18^. To coat membranes on CuB-Lips (M@CuB-Lips), we mixed membrane vesicles with CuB-Lips and coextruded the mixture through 0.40 μm and 0.22 μm porous polycarbonate membranes.

The liposomes’ hydrodynamic diameter and Zeta potentials were measured using a Nano particle size and Zeta potential analyzer (Benano 90 Zeta, Bettersize, China). The liposomes’ morphology was observed via transmission electron microscopy (TEM, Hitachi H-7650 C, Japan). To evaluate the retention of membrane proteins within M@CuB-Lips, an SDS-PAGE electrophoresis assay was performed, followed by staining with Coomassie brilliant blue. Examine the in vitro release behavior of liposomes using the dialysis method and detect the CuB release amount by HPLC (K2025, Hanon, China).

### In vitro cellular uptake of M@CuB-Lips

The uptake behavior of Cy5.5-loaded M@CuB-Lips or CuB-Lips by GL261 cells was examined using confocal laser scanning microscope (CLSM, Leica TCS SP5, Germany) and FCM. For CLSM, GL261 cells were seeded in 35 mm glass bottom dishes and treated with M@CuB-Lips or CuB-Lips for 1–2 h. The cells were then fixed, stained with DAPI, and examined under CLSM. For FCM, GL261 cells were seeded in 6-well plates, treated, prepared into a single-cell suspension, and detected by FCM.

A BBB culture model was constructed using bEnd.3 cells plated on the upper chambers of a transwell system (Corning, USA). Once the transendothelial electrical resistance exceeded 200 Ω·cm^2^, Cy5.5-loaded Lips were added to the upper chambers for 2, 4, or 8 h. The lower chamber’s culture medium was then collected for fluorescence detection.

### Distribution of M@CuB-Lips in mice

Cy5.5-loaded liposomes were injected intraperitoneally into mice with intracranial gliomas. After 24 h, the mice were euthanized and their organs collected. Fluorescent signal intensity in each organ was quantified using an IVIS system.

### Antitumor activity of M@CuB-Lips in vivo

C57BL/6 (female, 6–8 weeks old, 18–20 g) were purchased from Jinan pengyue laboratory animal breeding co.ltd (China). Mouse brain tumor model was established. On day 7, D-luciferin sodium salt was injected intraperitoneally, and mice were grouped based on IVIS fluorescence detection. The control group received blank liposomes intraperitoneally, while the M@CuB-Lips group and CuB group (0.5 mg/kg CuB) received daily intraperitoneal injections. On day 21, mice were then humanely euthanized by isofurane followed by cervical dislocation by cervical dislocation. 3 mice per group for brain tumor analysis and the rest for whole brain fixation with 4% paraformaldehyde. Tumors were digested into single-cell suspensions, blocked with CD16/32, and stained with primary antibodies (APC-CD3, PE-CD8, FITC-CD4, PE-Foxp3, Elabscience, China). Cells were analyzed. Brain tissue sections were stained with HE for morphology observation and photographed. Immunofluorescence was used to detect p-STAT3 expression in tumors, and HE staining was also performed on major tissue sections.

### Statistical analysis

Statistical analysis was performed using GraphPad Prism 8.0. The quantitative results were expressed as mean ± SD. P value less than 0.05 was considered statistically significant.

## Results

### CuB inhibits cell viability, colony formation and cell cycle distribution of GBM cells

**Fig. 1:**
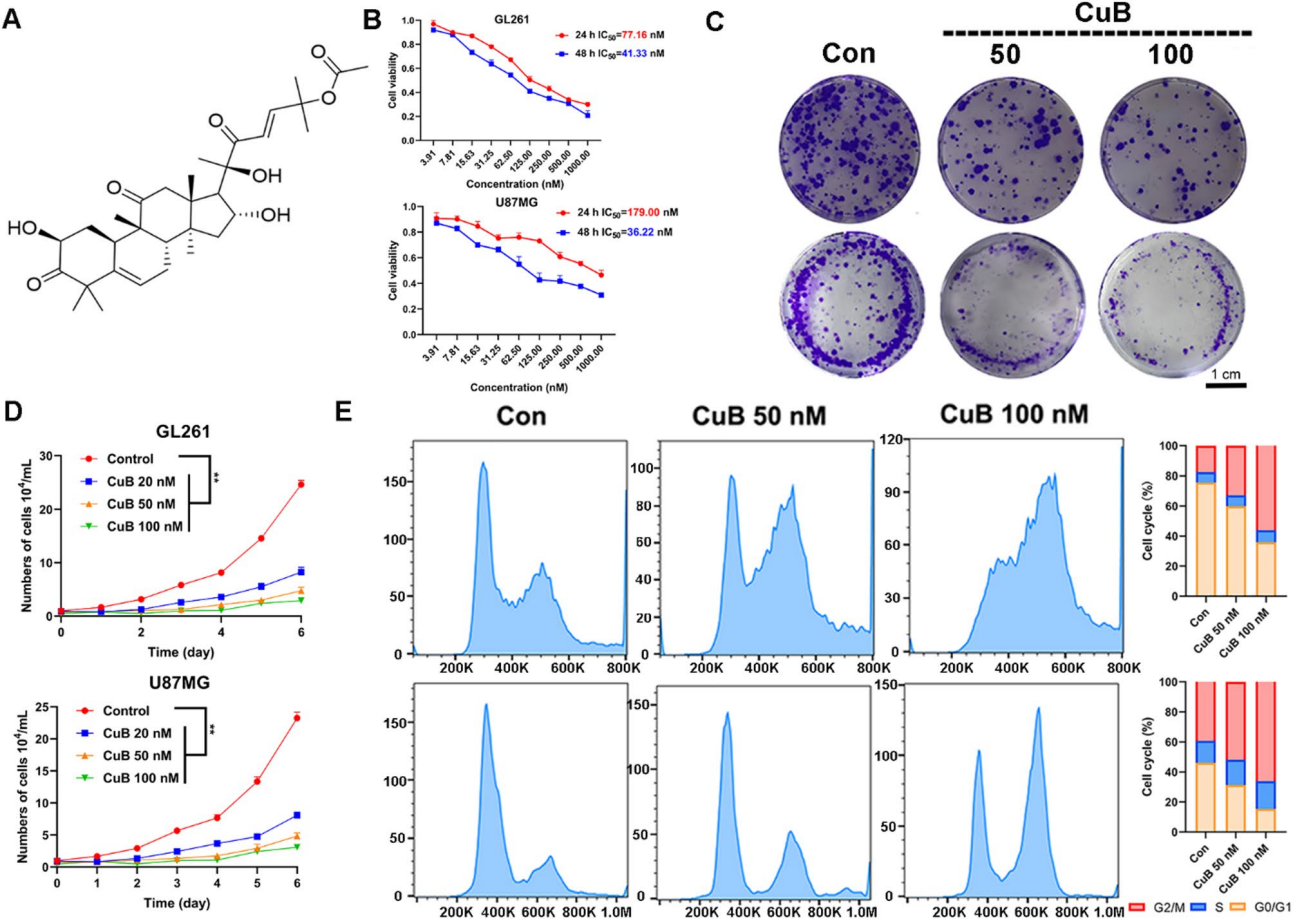
Antitumor activity of CuB in vitro. (**A**) CuB’s chemical structures. (**B**) GBM cell viability following 24, 48 h of CuB treatment, detected with the MTT assay, (**C**) Clone formation assay. GBM cells were exposed to 24 h of CuB treatment (50, 100 nM), before an additional 10 days of incubation, and representative experiments and images are shown. (**D**) Cell growth curves. GBM cells were grown for 6 days presence of indicated concentration of CuB (20, 50, 100 nM). (**E**) GBM cells received 24 h of CuB treatment (50, 100 nM). Then the cell cycle was analyzed through FCM. **p < 0.01.

In the present study, we investigated the anti-proliferative effects of CuB on GBM cell lines (GL261 and U87MG). The CuB structure is shown in Fig. 1A. Firstly, the effect of CuB on cell viability was assessed using the MTT assay. Specifically, the results indicated that CuB decreased the viability of GBM cells in a manner dependent on both time and dose. The IC_50_ values at 48 h were determined to be 41.33 nm for GL261 cells and 36.22 nm for U87MG cells, respectively (Fig. 1B), while exhibiting relatively low toxicity toward normal HT22 cells **(Figure ****S1****)**. The cell growth curves experiment supported this result (Fig. 1D). Furthermore, colony formation experiments demonstrated that CuB reduced cell growth and colony-forming capacity in a dose-dependent manner (Fig. 1C).

To further determine whether the anti-proliferative effects of CuB were associated with cell cycle arrest, flow cytometric analysis was performed. FCM assays revealed that, compared to control cells, the ratio of GBM cells in the G_2_/M phase significantly increased after 24 h of incubation with CuB (Fig. 1E). These findings indicate that CuB exerts anti-proliferative effects on GBM cells by arresting the cell cycle at the G_2_/M phase.

### CuB induces apoptosis via STAT3/ROS/ER stress pathway

**Fig. 2:**
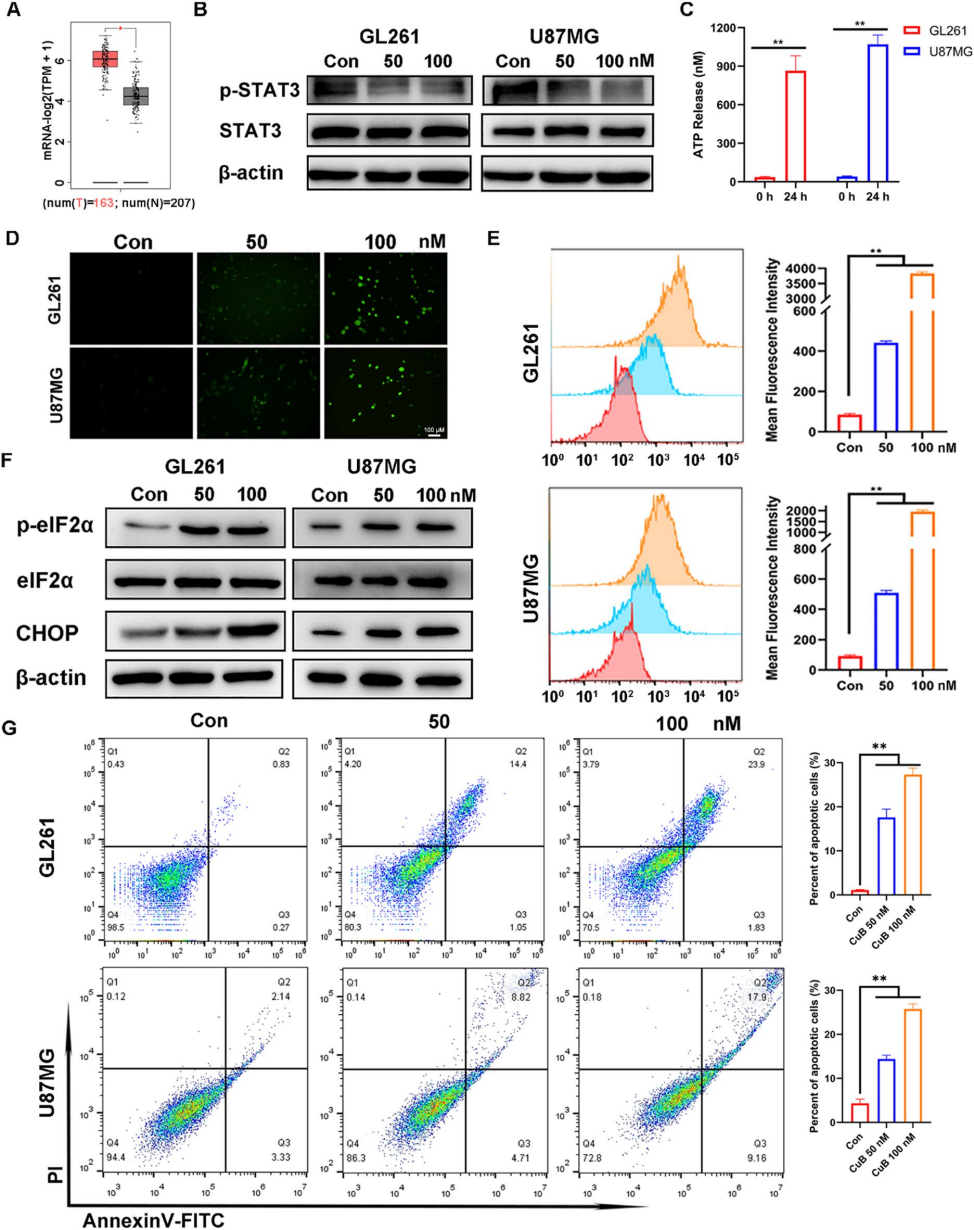
CuB induced apoptosis through the STAT3/ROS/ER Stress pathway. (**A**) Expression of STAT3 between GBM tissues and normal tissue was evaluated using GEPIA analysis. GBM cells received 24 h of CuB treatment (50, 100 nM). (**B**) Western Blotting assay of p-STAT3 and STAT3. (**C**) Extracellular ATP secretion detected by ATP-dependent liuminescence kit. (**D**, **E**) Intracellular ROS levels were detected by fluorescence microscopy and FCM. (**F**) Western Blot analysis of p-eIF2α, eIF2α and CHOP. (**G**) The apoptotic cells were detected by FCM. *p < 0.05. **p < 0.01.

We analyzed the expression of STAT3 in GBM using the GEPIA database (http://gepia.cancer-pku.cn/) and found that it was abnormally overexpressed compared to normal tissue (Fig. 2A). This suggests that STAT3 may be an important oncogene and therapeutic target for GBM. Here, we profiled the expression of STAT3 and p-STAT3 proteins in GBM cells. The Western blot results indicate that CuB can effectively inhibit the expression of p-STAT3(Fig. 2B, S2). Simultaneously, a significant release of ATP is also observed (Fig. 2C), which is indicative of cellular stress. Moreover, ATP serves as one of the key hallmarks for the occurrence of immunogenic cell death (ICD) in cells^19^. Studies have shown that STAT3 is crucial for the synthesis of glutathione (GSH). Blocking STAT3 can significantly reduce GSH levels and increase ROS levels, leading to oxidative DNA damage in cells^20^. To quantitatively measure changes in ROS production. we used DCFH-DA dye staining after treatment with CuB for 24 h. The fluorescence signal of DCF in the CuB group was much greater than that in the Con group, confirming that CuB effectively induced an increase in ROS levels in GBM cells (Fig. 2D, E). Excessive oxidative stress has the potential to disrupt the function of redox-sensitive organelles, among which the endoplasmic reticulum (ER) is particularly vulnerable. As it is well-known, ER stress plays a significant role in promoting apoptosis^21^. In order to delve into whether CuB has the ability to induce ER stress in GBM cells, we initiated our investigation by assessing the activities of eIF2α-CHOP ER stress sensor proteins in GBM cells that had been incubated with CuB. The results of the Western blot experiments revealed a significant elevation in the levels of p-eIF2α and CHOP proteins in GBM cells after incubation with CuB, compared to the control group (Fig. 2F, S3). This indicates that CuB activates the ER stress pathway, which is an important pathway for tumor apoptosis and ICD^22^. To confirm ICD induction, we detected key damage-associated molecular patterns (DAMPs): cell surface exposure of calreticulin (CRT) and release of high-mobility group box 1 (HMGB1). Our results demonstrated that CuB treatment significantly promoted CRT expose (**Figure. S4**) and increased extracellular HMGB1 release (**Figure. S5**) Furthermore, we employed Annexin V-FITC/PI double staining to examine the effect of CuB on apoptosis in GBM cells. The results demonstrated that CuB could induce apoptosis in GBM cells in a dose-dependent manner (Fig. 2G). The above results indicate that CuB induces an increase in ROS levels by inhibiting the expression of p-STAT3, which in turn stimulates the activation of the endoplasmic reticulum stress pathway, leading to apoptosis in GBM cells.

### CuB inhibits the growth of GBM xenograft in vivo

**Fig. 3:**
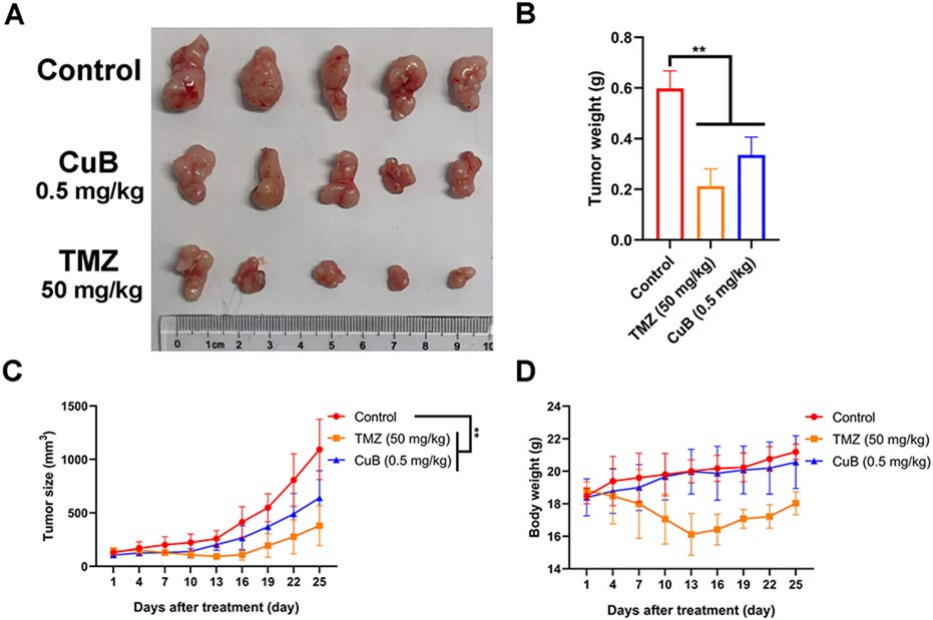
CuB (0.5 mg/kg/day) inhibited GBM growth in xenograft mouse model. (**A**) The typical tumor images of different groups. (**B**) Tumor weight, (**C**)Tumor size and (**D**) Body weight were measured. **p < 0.01.

We then investigated the in vivo anti-tumor efficacy of CuB in a U87MG xenograft glioma model. The findings demonstrated that xenograft tumors formed from U87MG cells might be inhibited in their growth by CuB at dosages of 0.5 mg/kg. 45% inhibition rate was found (Fig. 3A-C). We also observed that the body weight in the animals treated with TMZ, but not CuB, were significantly decreased with controls (Fig. 3D). These results suggest that CuB may be a low-toxicity, high-efficiency candidate compound for CuB treatment. However, the application of CuB in GBM treatment is limited due to its poor water solubility and the presence of the BBB. Thus, how to efficiently and accurately deliver CuB to brain tumor sites is an important issue that needs to be addressed.

### Preparation, characterization and targeting evaluation of M@CuB-Lips

**Fig. 4:**
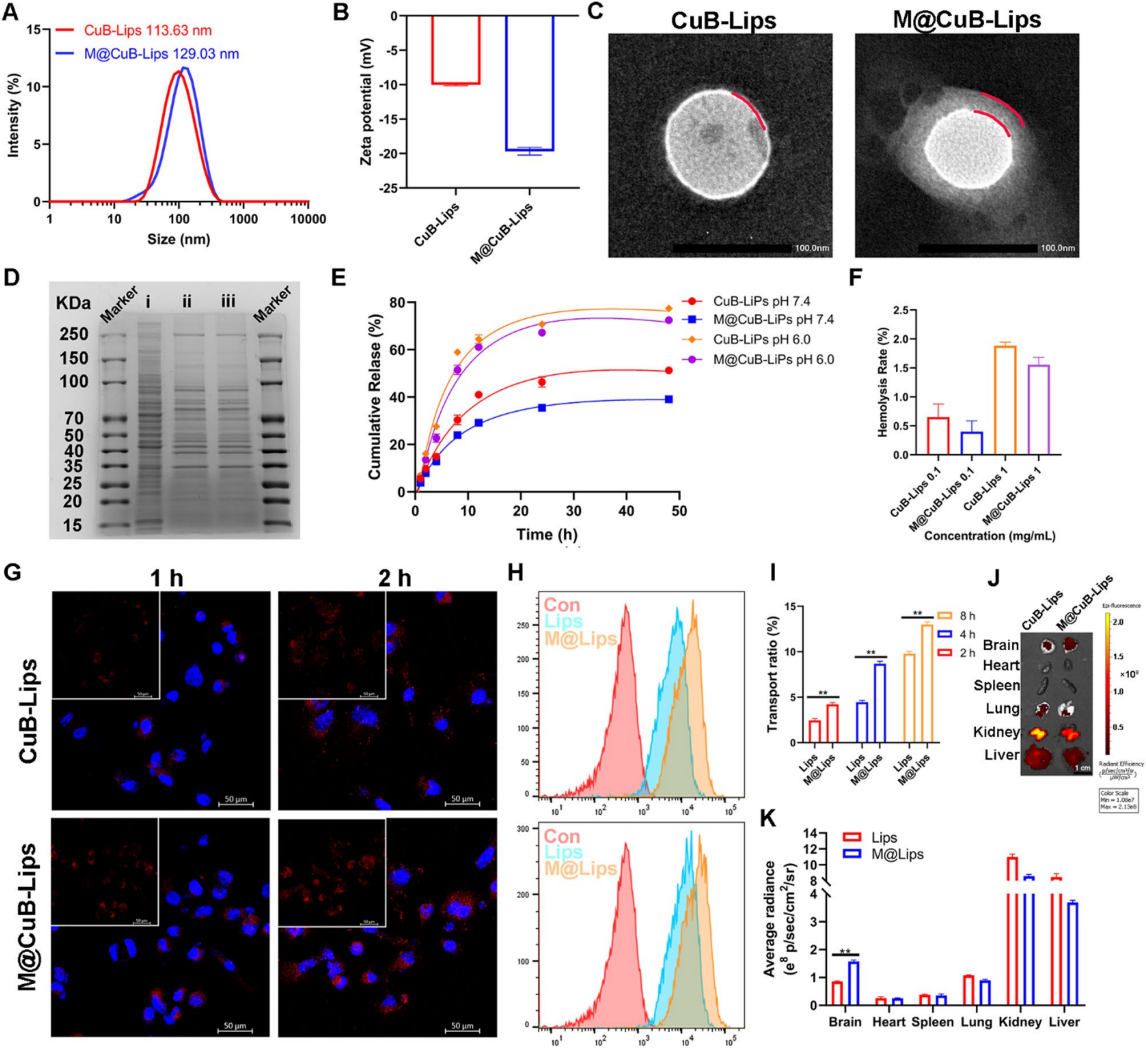
Characterization and targeting of M@CuB-Lips. (**A**, **B**) Size and zeta-potential measured through DLS. (**C**) TEM images. (**D**) Protein profiles of GL261(I), GL261 membrane (II), and M@CuB-Lips (III). (**E**) The release curves of CuB under different acidic environments (pH 6.0 or 7.4). (**F**) The hemolysis rate of M@CuB-Lips in vitro. (**G**, **H**) The GL261 cells uptake analyzed by CLSM and FCM. (**I**) The penetration efficiency of M@CuB-Lips analyzed by a BBB model in vitro. (**J**, **k**) Distribution and analysis of M@CuB-Lips in GBM mice. **p < 0.01.

We prepared CuB liposomes(CuB-Lips)for the loading of CuB. Furthermore, we utilized GL261 cell membranes to encapsulate CuB-Lips༈M@CuB-Lips༉for homologous targeting of GBM tumors. The hydrodynamic size and distribution of CuB-Lips and M@CuB-Lips were analyzed using dynamic light scattering (DLS). CuB-Lips exhibited a narrow unimodal distribution, with a z-averaged diameter of approximately 113.63 nm. In contrast, Ang-M@CuB-Lips had a z-averaged diameter of 129.03 nm (Fig. 4A). The surface of CuB-Lips was negatively charged, possessing a zeta-potential of -10.00 mV, whereas the zeta-potential of M@CuB-Lips decreased to -19.67 mV (Fig. 4B). Transmission electron microscopy (TEM) observations revealed that both CuB-Lips and M@CuB-Lips possessed spherical morphologies. Notably, a film was observed on the surface of M@CuB-Lips (Fig. 4C). The electrophoretic bands also confirm that the cell membrane is coated on the outer layer of Lips (Fig. 4D). The in vitro release results indicate that CuB can be effectively released from M@CuB-Lips under acidic conditions (Fig. 4E). Hemolysis experiment showed that M@CuB-Lips had a good biosafety (Fig. 4F).

Furthermore, we investigated the uptake of Cy5.5 loaded M@CuB-Lips by GBM cells using CLSM and FCM (Fig. 4G, H). The results clearly demonstrated that M@CuB-Lips achieved a higher intracellular delivery of Cy5.5 into GL261 cells compared to uncoated CuB-Lips. Through in vitro BBB model experiments, M@CuB-Lips exhibits stronger BBB permeability compared to CuB-Lips (Fig. 4I). Similarly, tissue distribution experiments also confirm that M@CuB-Lips has enhanced accumulation capability in brain tumors, in comparison to CuB-Lips (Fig. 4J, K).

### M@CuB-Lips inhibits the growth of GBM in vitro and in vivo

**Fig. 5:**
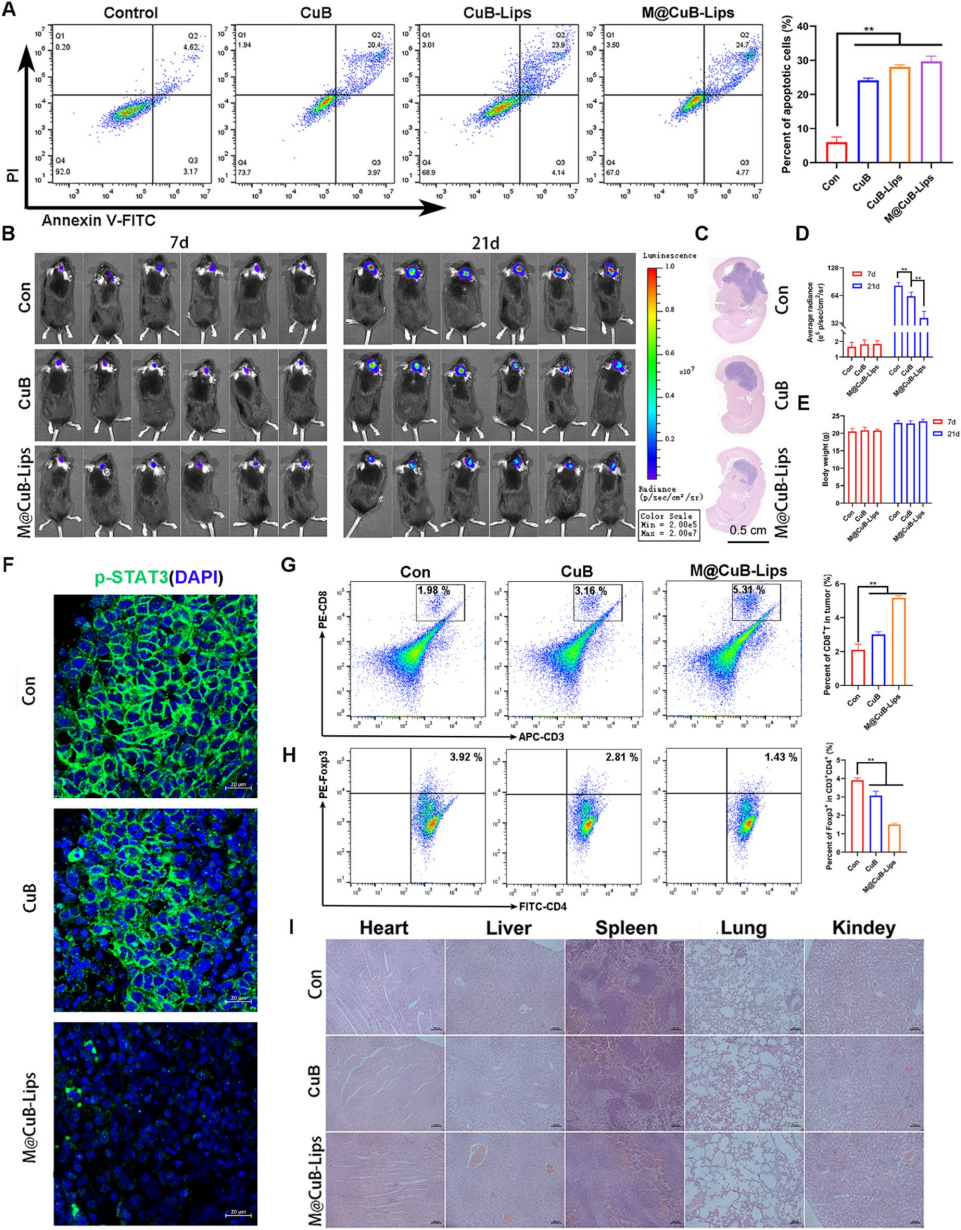
M@CuB-Lips inhibits GBM growth in vitro and in vivo. (**A**) GBM cells were treated with M@CuB-Lips, CuB-Lips or CuB (100 nM) for a 24-h duration. Cell apoptosis was detected through FCM. (**B**, **D**) Inhibition of M@CuB-Lips or CuB on the orthotopic GBM observed by IVIS. (**C**) HE staining images of the brain slices. (**E**) Body weight of mice. (**F**) Immunofluorescence images of p-STAT3 within tumor. (**G**) CD8^+^ cell proportion (gated on CD3^+^ CD8^+^ cells) within tumor. (**H**) Treg cell proportion (gated on CD3^+^CD4^+^Foxp3^+^ cells) in tumor. (**I**) HE staining images for liver, heart, spleen, lung, and kidney slices. **p < 0.01.

By employing Annexin V-FITC/PI double staining, we investigated the impact of M@CuB-Lips on apoptosis in GBM cells (Fig. 5A). The results demonstrated that M@CuB-Lips exhibited potent apoptosis-inducing capabilities, surpassing those of CuB. Subsequently, we established an orthotopic brain tumor model using Luc^+^-GL261 cells. Experimental outcomes revealed that, following 14 days of treatment, M@CuB-Lips effectively inhibited the proliferation of GL261 cells at their orthotopic site in the brain, with a significantly greater therapeutic efficacy compared to CuB (Fig. 5B-D). Immunofluorescence analysis further indicated that M@CuB-Lips markedly downregulated the levels of p-STAT3 protein at the tumor site within the brain (Fig. 5F).

Additionally, we examined the immune cells within the brain tumor. The results showed that M@CuB-Lips effectively increased the infiltration of CD8^+^ T cells in the brain tumor while reducing the levels of Treg cells (Fig. 5G, H). This would contribute to ameliorating the immunosuppressive microenvironment of brain tumors. After treatment with M, there was no significant difference in body weight between the treated mice and the control group (Fig. 5E). Pathological examinations of major organs in mice revealed no damaging effects of M@CuB-Lips on these organs (Fig. 5I). Collectively, these findings suggest that CuB is a safe and effective drug for inhibiting GBM, and the formulation of M@CuB-Lips further enhances its application in orthotopic brain tumors.

## Discussion

GBM, as a malignant tumor, poses a severe threat to human health. The obstacle posed by the BBB hinders the direct delivery of most drugs to the tumor site. Additionally, its unique and cold heterogeneous tumor microenvironment leads to drug resistance to many chemotherapeutics and poor efficacy of immunotherapy^23^. Therefore, an in-depth exploration of the molecular information of GBM to identify novel and effective therapeutic agents and ensure their precise action at the tumor site represents a crucial research direction in GBM treatment.

CuB, a tetracyclic triterpenoid compound, has been isolated from Cucumis melo of the Cucurbitaceae family. Previous studies have demonstrated its potent antitumor activity against various tumors^24–26^. In our current research, we investigated its inhibitory effect on the proliferation of GBM cells. The results of in vitro proliferation inhibition experiments indicated that CuB can effectively suppress the proliferation and colony formation of GBM cells. Further studies revealed that CuB efficiently arrests the GBM cell cycle at the G_2_/M phase, consistent with its effects observed in other tumor types. The underlying mechanism may involve its impact on tubulin, similar to its mode of action in other tumors. Furthermore, we established a subcutaneous xenograft tumor model of GBM to evaluate the antitumor activity of CuB. The results demonstrated that CuB significantly inhibited the in vivo proliferation of GBM cells at a very low concentration of 0.5 mg/kg, without exerting any adverse effects on the body weight of the mice. Therefore, CuB may represent an efficacious and low-toxicity therapeutic agent for GBM.

To explore the mechanism underlying the antitumor effects of CuB on GBM, we conducted a comprehensive investigation of the molecular biological profile of GBM. Our findings revealed a high expression of STAT3 in GBM, and the aberrant activation of STAT3 may be a crucial factor contributing to excessive proliferation of GBM, chemotherapy resistance, as well as the immunosuppressive tumor microenvironment^27^. Consequently, we validated the impact of CuB on STAT3 through Western blot experiments, and found that CuB effectively inhibited the phosphorylation of STAT3 in GBM cells. This phosphorylation is a necessary condition for the function of STAT3 protein. STAT3 serves as a key transcriptional factor in regulating tumor energy metabolism. Studies have indicated that blocking STAT3 may lead to an explosive increase in ROS^20^. Therefore, we employed the fluorescent probe DCFH-DA to assess the impact of CuB on ROS levels in GBM cells. Consistent with our expectations, the results demonstrated a significant elevation in ROS levels. Excessive ROS can exert various deleterious effects, potentially disrupting the function of redox-sensitive organelles, including the ER. Therefore, we evaluated the impact of CuB on the ER in GBM cells by examining the endoplasmic reticulum stress protein eIF-2α/CHOP pathway. The experimental results demonstrated that CuB activated endoplasmic reticulum stress, which can induce apoptosis. Flow cytometry analysis further confirmed the occurrence of apoptosis. Based on these findings, we conclude that CuB upregulates ROS levels in GBM cells by inhibiting STAT3 phosphorylation, subsequently triggering excessive ER stress and inducing cell apoptosis. This aligns with CuB’s known mechanism of action in other cancers where it directly binds and inhibits STAT3 phosphorylation^11,28^, triggering downstream effects like ROS elevation and ferroptosis^10^.

In the context of therapeutic agents for GBM, accurately delivering drugs across the BBB to target brain tumors poses a critical challenge that must be addressed. The EPR effect, a phenomenon where nanoparticles preferentially accumulate in tumor tissues due to their leaky vasculature and poor lymphatic drainage, has been widely exploited in cancer nanomedicine. Cancer cell membrane-coated liposomes, such as M@CuB-Lips developed in this study, leverage not only the EPR effect but also the homologous targeting ability derived from the cancer cell membrane. The homologous targeting ability of cancer cell membrane-coated nanocarriers has been documented in several studies^29^. Similarly, our study observed enhanced uptake of M@CuB-Lips by GBM cells compared to uncoated liposomes, indicating the effectiveness of homologous targeting (Fig. 4G, H).

In vitro uptake experiments demonstrated that GL261 cells exhibited favorable absorption of the liposomes. Notably, due to the homologous targeting property of cellular membranes, CL261 cells showed a higher uptake of M@CuB-Lips. To evaluate the BBB crossing capability of M@CuB-Lips, we established an in vitro BBB model using bEnd.3 cells. Experimental results indicated that M@CuB-Lips exhibited excellent penetration ability through the in vitro BBB model. Furthermore, we investigated the tissue distribution of M@CuB-Lips in mice with orthotopically implanted brain tumors. Consistent with the in vitro findings, a significant accumulation of M@CuB-Lips was observed in the brain, collectively proving the ability of M@CuB-Lips to cross the BBB and deliver to brain tumors.

We evaluated the in vivo inhibitory effects of M@CuB-Lips on GBM using an orthotopic brain tumor model. Experimental results demonstrated that M@CuB-Lips exhibited robust inhibitory effects on orthotopically implanted brain tumors, with an inhibition rate exceeding 60% after 14 days of treatment. Immunofluorescence analysis revealed that M@CuB-Lips significantly suppressed the expression level of p-STAT3 in the tumor site, which may represent a crucial mechanism of CuB’s antitumor activity against GBM. The abnormal activation of STAT3 contributes to an immunosuppressive tumor microenvironment in GBM, leading to inadequate infiltration of cytotoxic immune cells^27^. This may account for the cold tumor microenvironment in GBM and the ineffectiveness of immunotherapy in this disease. The inhibitory effect of CuB on p-STAT3 may optimize the immune environment of GBM, and concurrently, CuB’s impact on the ER of GBM cells may induce ICD, further enhancing the immunogenicity of GBM. Therefore, we investigated the presence of CD8^+^ T cells and Treg cells in the GBM tumor site and found that M@CuB-Lips effectively increased the infiltration of CD8^+^ T cells and inhibited Treg cells in the tumor. These findings indicate that CuB not only serves as a direct cytotoxic agent against GBM but also has potential as an immunotherapeutic agent for improving the cold immune microenvironment of GBM.

After 14 days of treatment, neither CuB nor M@CuB-Lips had a significant impact on the body weight of the mice. Additionally, histological examination using HE staining of major organs (heart, liver, spleen, lungs, and kidneys) revealed no apparent pathological changes. These findings suggest that CuB is a safe therapeutic agent for the treatment of GBM. Notably, immune regulators such as DC maturation status, myeloid-derived suppressor cells (MDSCs), and macrophage polarization also play critical roles in shaping the immunosuppressive GBM niche. Future studies will focus on elucidating CuB’s regulatory effects on these immune modulators, which could further expand its potential as a multifaceted immunotherapeutic strategy for GBM treatment.

## Conclusion

**Fig. 6:**
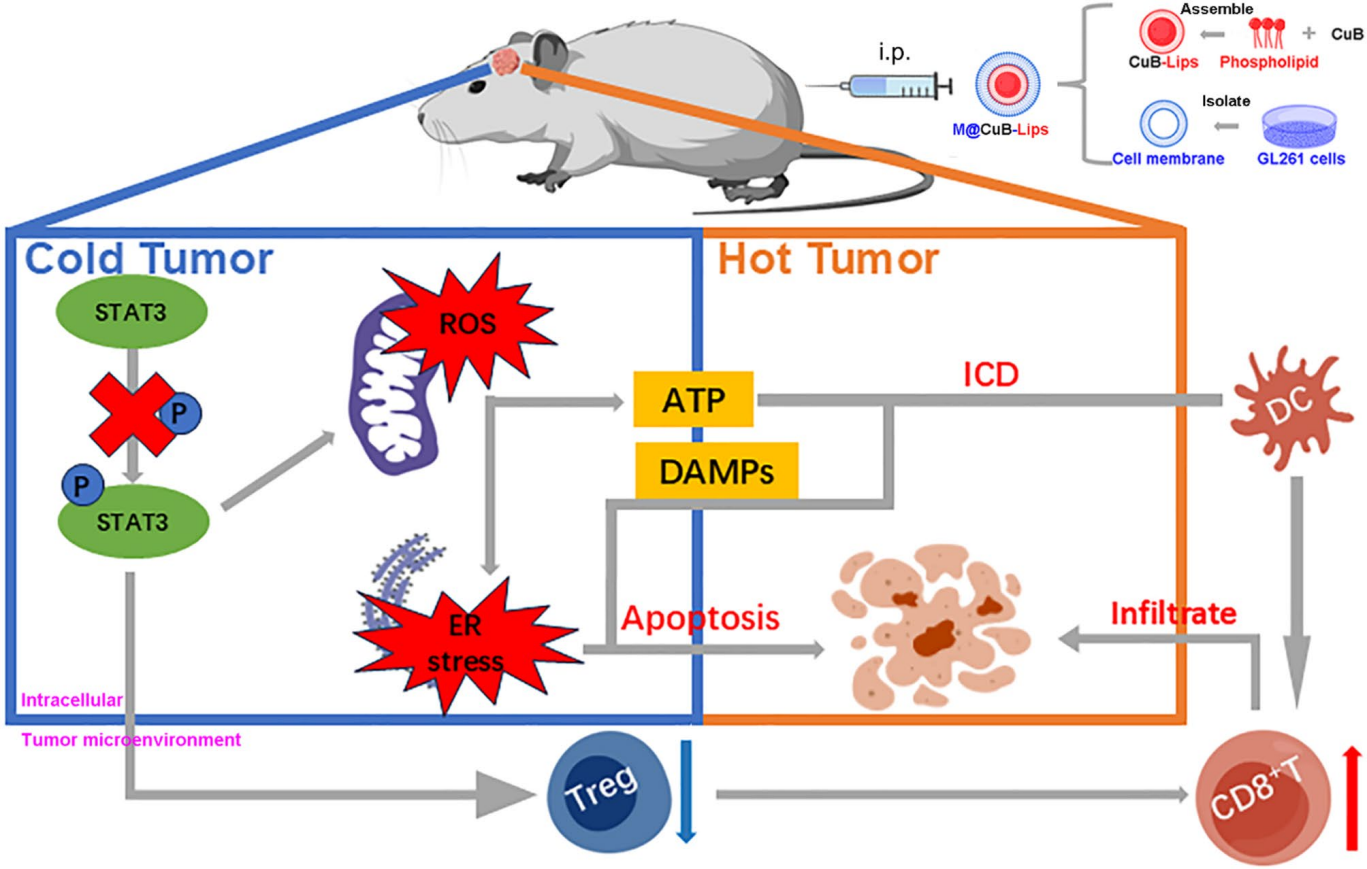
Schematic diagram depicting the underlying mechanisms of M@CuB-Lips against GBM growth. M@CuB-Lips achieve homologous targeting to GBM cells and penetrate the BBB. Upon intracellular delivery, CuB inhibits STAT3 phosphorylation, leading to elevated ROS levels and activation of the ER stress pathway, ultimately inducing tumor cell apoptosis. Concurrently, CuB-triggered ICD promotes the release of DAMPs, which enhance CD8^+^ T cell infiltration and cytotoxicity against GBM, thereby remodeling the immunosuppressive tumor microenvironment. Created with BioGDP.com^30^.

In conclusion, CuB exhibits significant antiproliferative and pro-apoptotic impacts on GBM, both in vitro and in vivo, consistent with its established efficacy in other malignancies. The novel M@CuB-Lips formulation overcomes key therapeutic barriers—including poor water solubility and BBB penetration—by leveraging homologous tumor-targeting properties derived from cancer cell membrane coating. This strategy significantly enhances site-specific drug delivery to brain tumors, offering a promising therapeutic approach for GBM. Furthermore, M@CuB-Lips demonstrates immunomodulatory potential by increasing CD8^+^T cell infiltration and suppressing Treg cells, thereby ameliorating the immunosuppressive tumor microenvironment. Mechanistically, CuB’s antitumor activity involves inhibition of STAT3 phosphorylation, which elevates ROS levels and activates the ER stress pathway (eIF2α/CHOP), ultimately inducing GBM cell apoptosis (Fig. 6).

## Supplementary Information

Below is the link to the electronic supplementary material.

Supplementary Material 1

## Data Availability

The data generated in the present study may be requested from the corresponding author.
