# Supplementary material for: Cucurbitacin B suppresses glioblastoma via the STAT3/ROS/endoplasmic reticulum stress pathway
Source: Sci Rep. 2025 Oct 27;15:37485. doi: 10.1038/s41598-025-21526-0 (PMC12559341; doi:10.1038/s41598-025-21526-0)
Supplement: Supplementary file 1 — Supplementary Material 1 [file 41598_2025_21526_MOESM1_ESM.docx]

**Methods**

**Fabrication of** **M@CuB-Lips**

CuB-Lips were prepared using a film dispersion method. Briefly, CuB (10 mg), lecithin (120 mg) and cholesterol (18 mg) were dissolved in methanol. After 5 min of stirring, the organic solvent was evaporated on a rotary evaporator under reduced pressure at 40˚C to obtain a membrane. The resulting membrane was dissolved by the addition of PBS (pH7.4) to obtain the CuB-Lips solution.

GL261cells were resuspended in pre-chilled TM buffer (10 mM Tris + 1 mM MgCl₂, pH 7.4) at a density of 3.0 × 10⁷ cells/mL. A 1% volume of PMSF was added to the cell suspension, followed by overnight hypotonic incubation at 4 °C to disrupt the plasma membrane structure. The suspension was then homogenized using a Dounce homogenizer with 30 repeated strokes to prepare the cell homogenate. The homogenate was mixed with 1 M sucrose solution to achieve a final sucrose concentration of 0.25 M. After centrifugation at 2,000 rcf for 15 min at 4 °C, the supernatant was collected while discarding the pellet to remove cellular debris, apoptotic bodies, and shed vesicles. The supernatant was further centrifuged at 3,000 rcf for 30 min, and the resulting pellet containing plasma membranes was collected.

To obtain M vesicles, the modified membrane underwent a process of extrusion ten times through polycarbonate porous membranes with pore sizes of 0.40 μm and 0.22 μm, using an Avestin mini extruder (Avestin, LF-1, Canada). To coat M on CuB-Lips, we mixed the M vesicles and CuB-Lips and then coextruded them with 0.40 μm and 0.22 μm polycarbonate porous membrane.

**Orthotopic glioma model in mice**

To establish an orthotopic glioma model in mice, we utilized a mouse brain stereotaxic apparatus. Using the lambda suture as the zero point, we positioned the target site at 2.5 mm to the right and 1.5 mm anterior, targeting the striatum region of the mouse brain. After marking the site, we used a drill to create a hole in the mouse skull. Taking the skull surface level as the zero point, we inserted the needle to a depth of 3.5 mm. A microinjection pump was then used to steadily inject 2 × 10⁵ GL261-Luc cells in 4 μL at a constant rate of 0.8 μL/min into the mouse brain, thereby establishing the orthotopic brain tumor model.

**CRT Immunofluorescence Staining**

GL261 or U87MG cells were treated with CuB for 6 h. After fixed with methanol, permeabilized with 0.5% Triton-X 100 and blocked by using 5% goat serum in PBS, cells were treated with anti-Calreticulin-AF488 antibody (1:400, ab196158, Abcam, MA, USA) overnight at 4 ℃. Nucleus was stained with DAPI. The exposure of CRT was visualized by confocal laser scanning microscopy (CLSM, Leica TCS SP5, Germany).

**HMGB1 Measurement**

GL261 or U87MG cells (5 × 10^4^ per well) seeded into 6-well plate with a cover were incubated with CuB (100 nM) for 24 h. For ELISA analysis, the supernatant collected from cells after treated and quantified by HMGB1 ELISA Kit (Elabscience,Wuhan, China).

**Results**


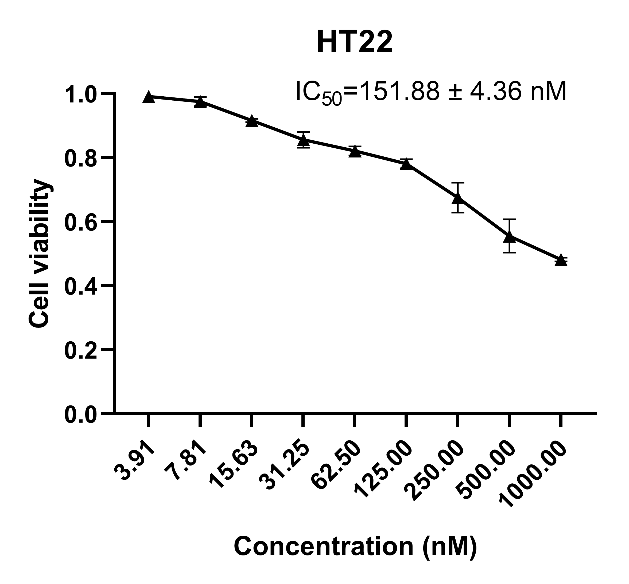


**Figure S1**. HT22 cell viability following 48 h of CuB treatment, detected with the MTT assay.


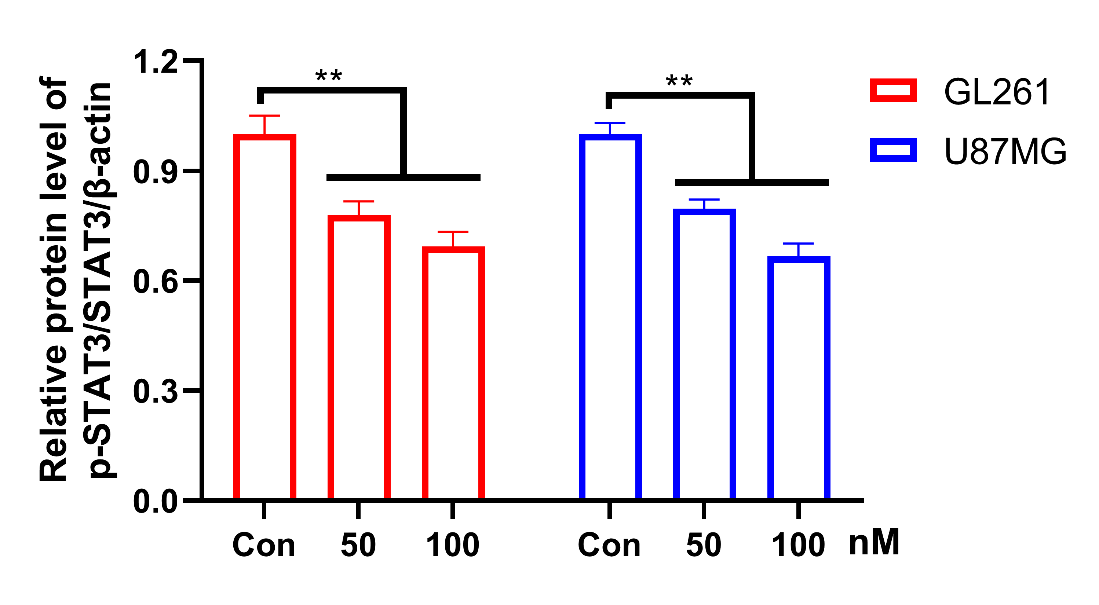


**Figure S2**. Relative protein level of p-STAT3/STAT3/β-actin following 24 h of CuB treatment. ***p* < 0.01.


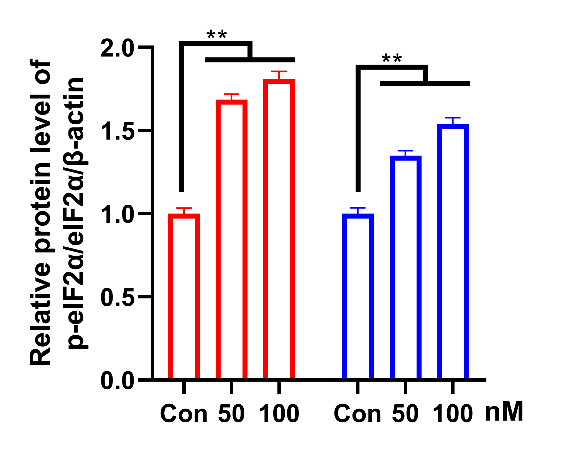

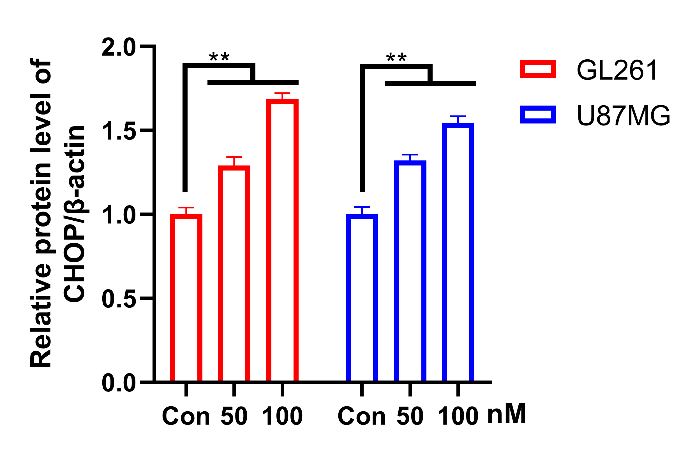
 **Figure S3**. Relative protein level of p-eIF2α/eIF2α/β-actin and CHOP/β-actin following 24 h of CuB treatment. ***p* < 0.01.

**
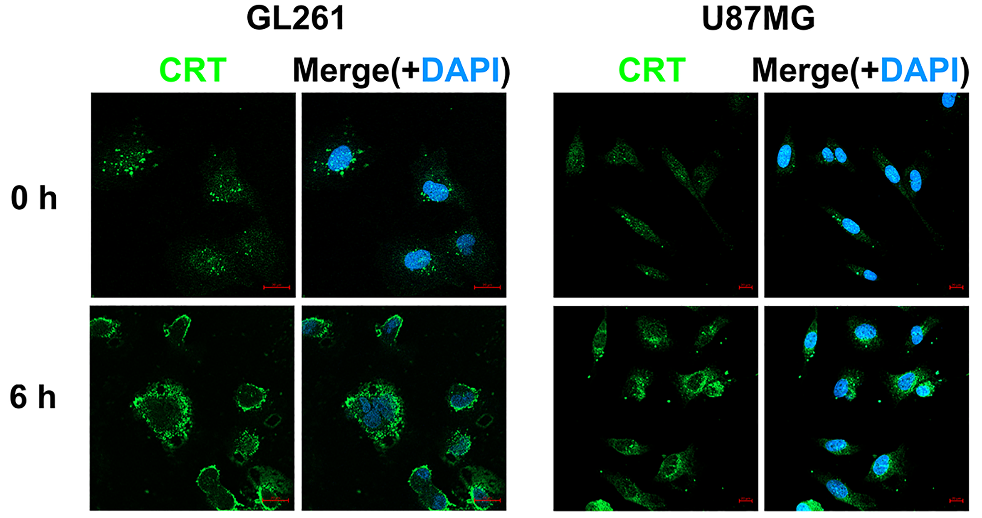
**

**Figure S4**. Immunofluorescence images of exposed CRT in CuB-treated cells.


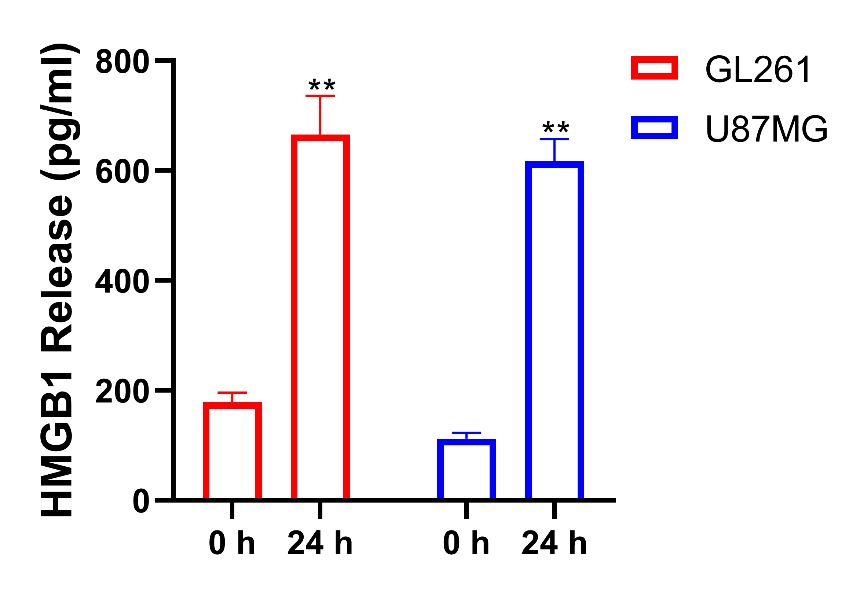


**Figure S5**. Measurement of release HMGB1 by ELISA following 24 h of CuB treatment. ***p* < 0.01.

**Western Blot**

**Figure 2B：**

**
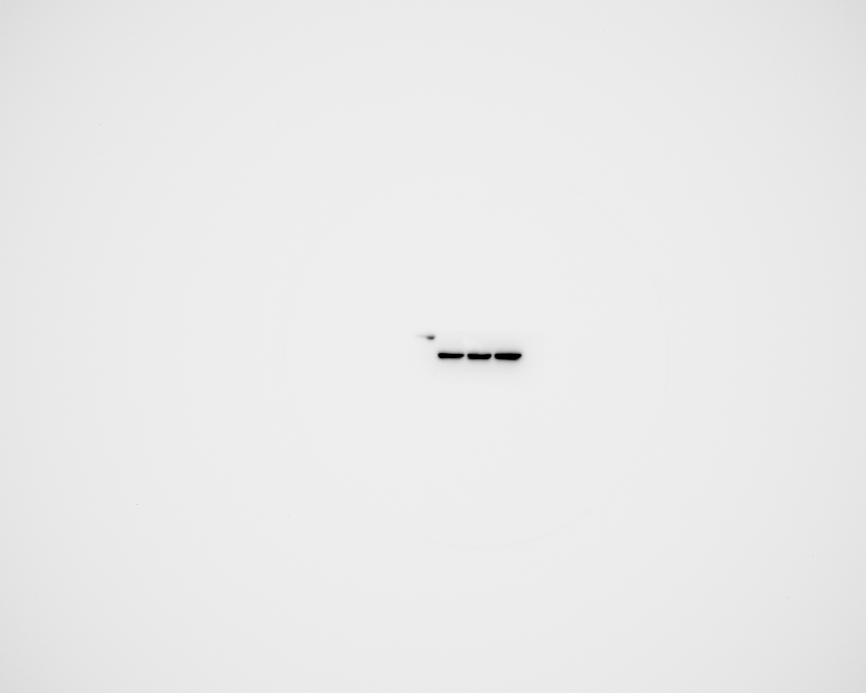
GL261 β-actin**

**GL261 STAT3**

**
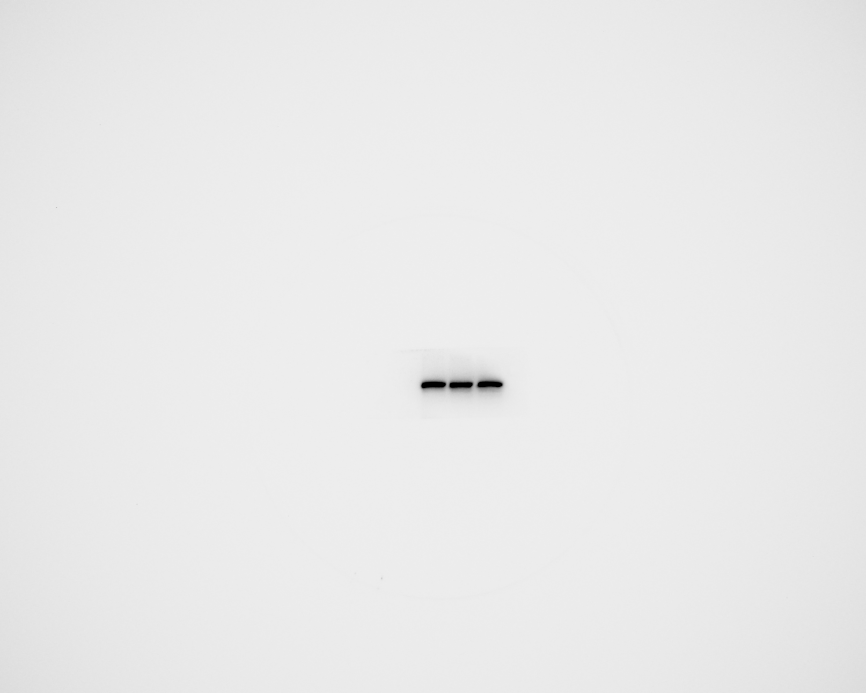
**

**
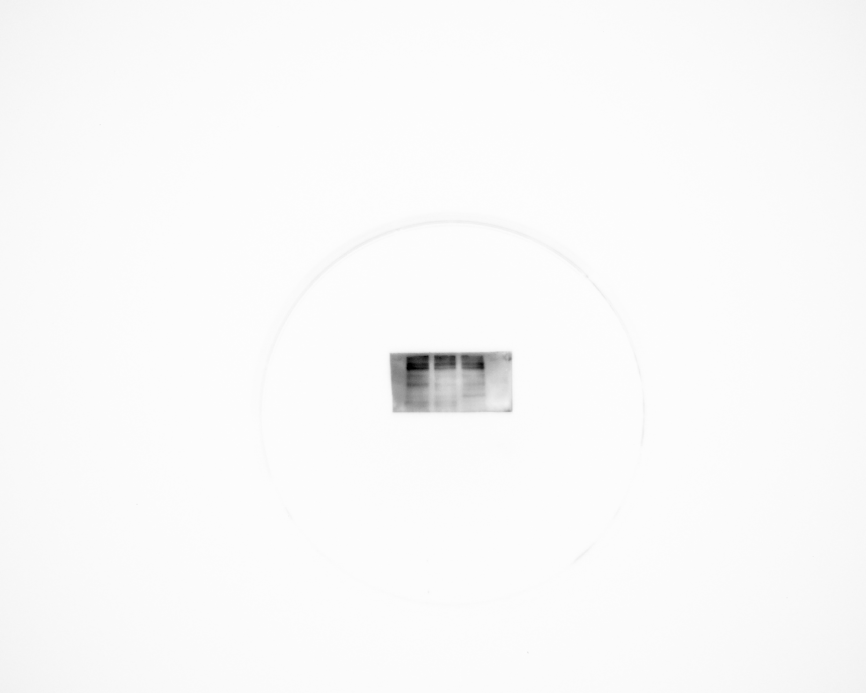
GL261 p-STAT3**

**U87MG β-actin**

**
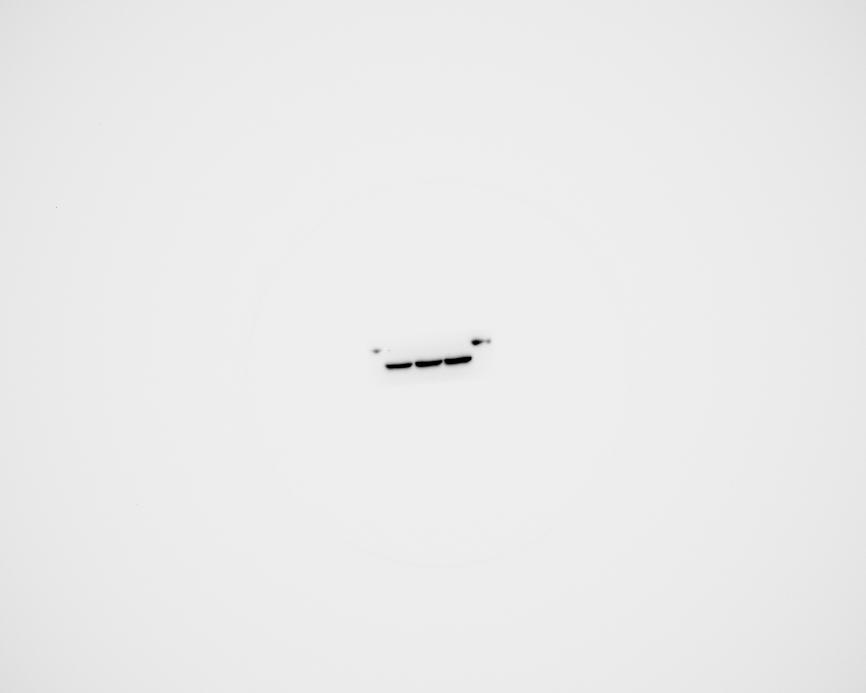
**

**U87MG STAT3**

**
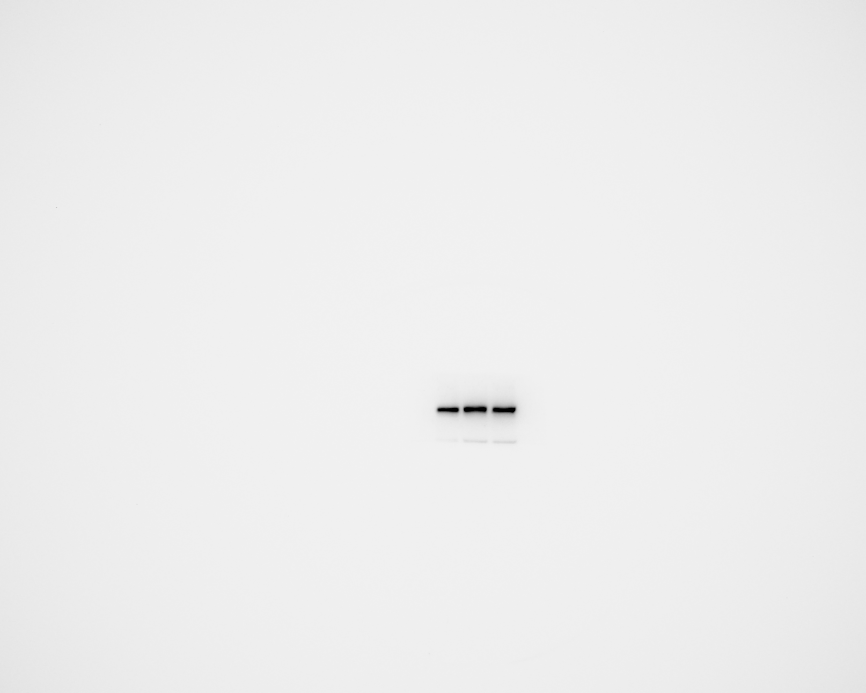
**

**U87MG p-STAT3**

**
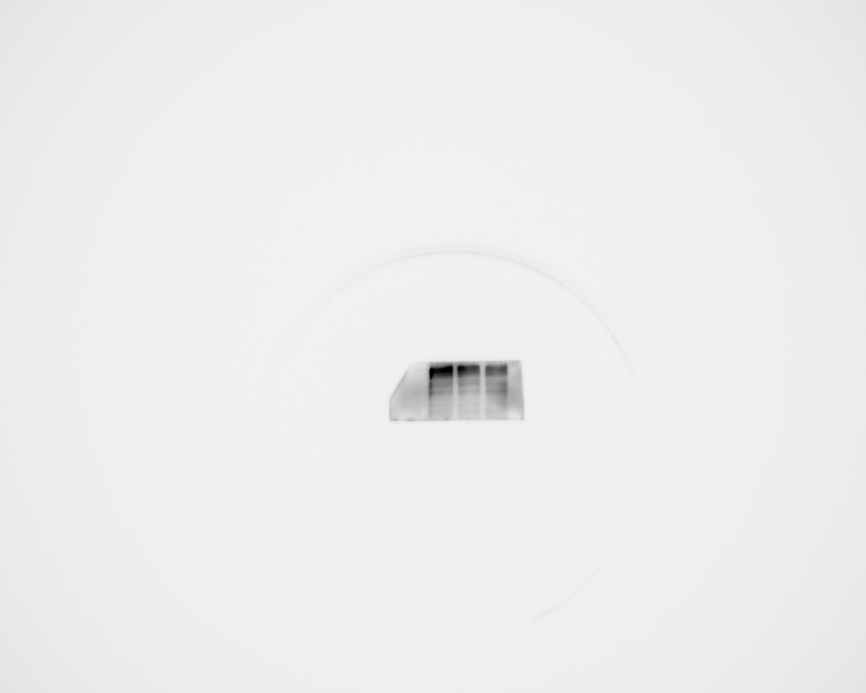
**

**Figure 2F**

**GL261 β-actin**

**
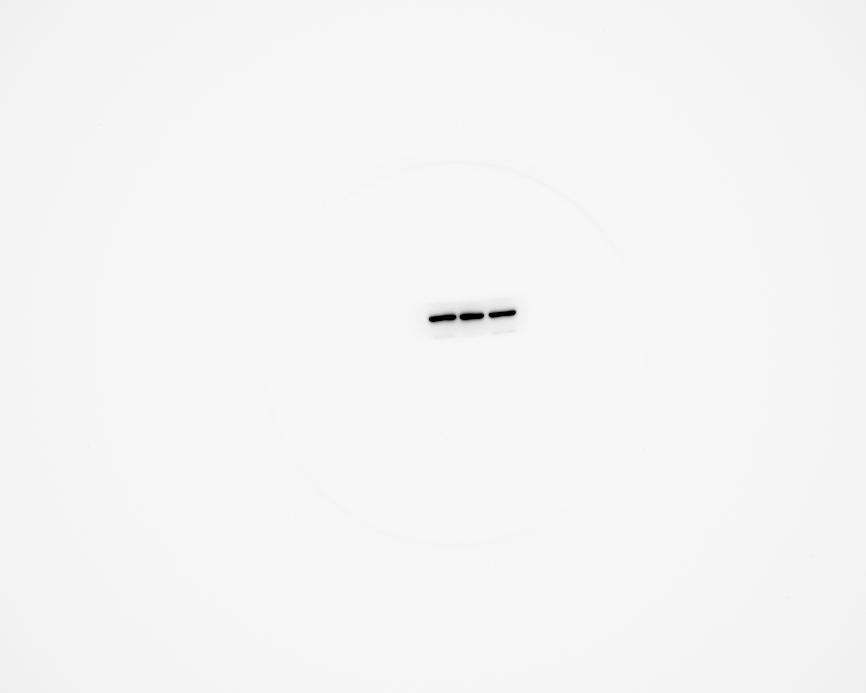
**

**GL261 CHOP**

**
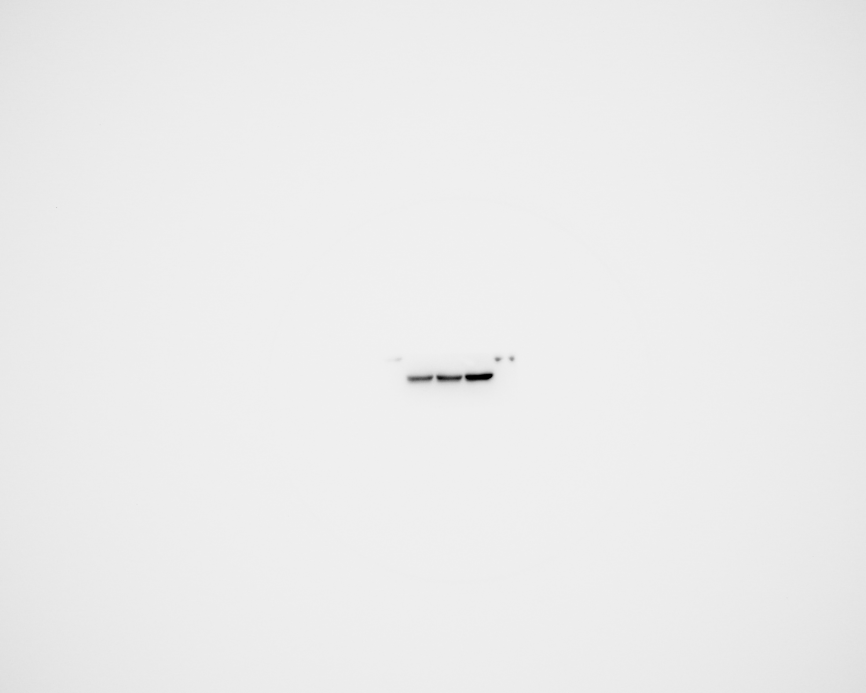
**

**GL261 eIF2α**

**
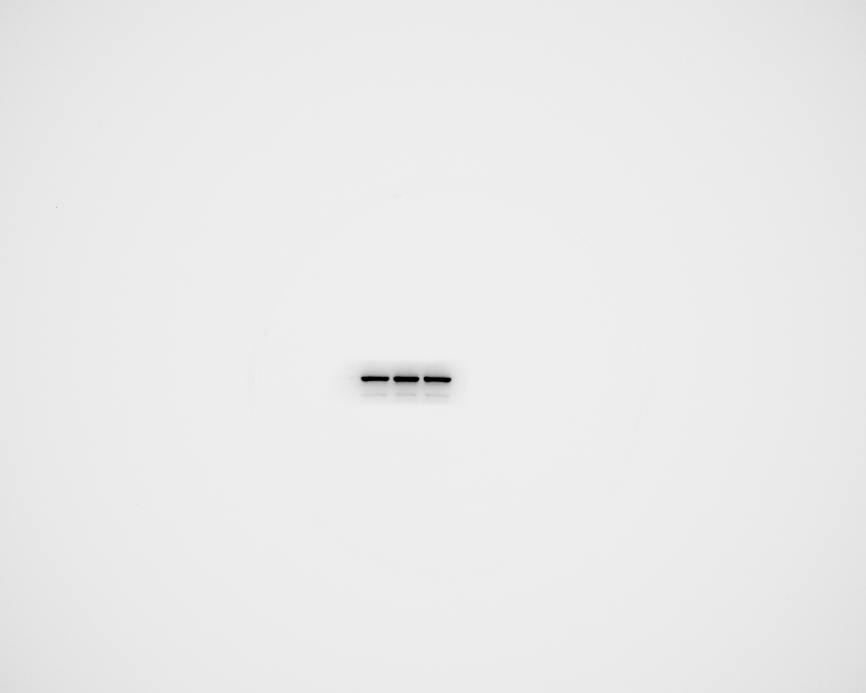
**

**GL261 p-eIF2α**

**
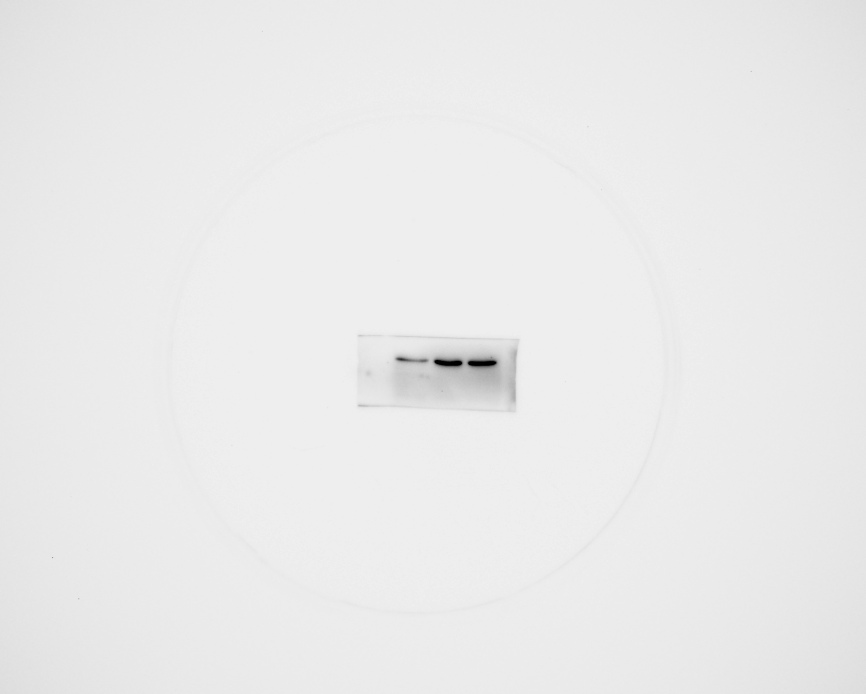
**

**U87MG β-actin**

**
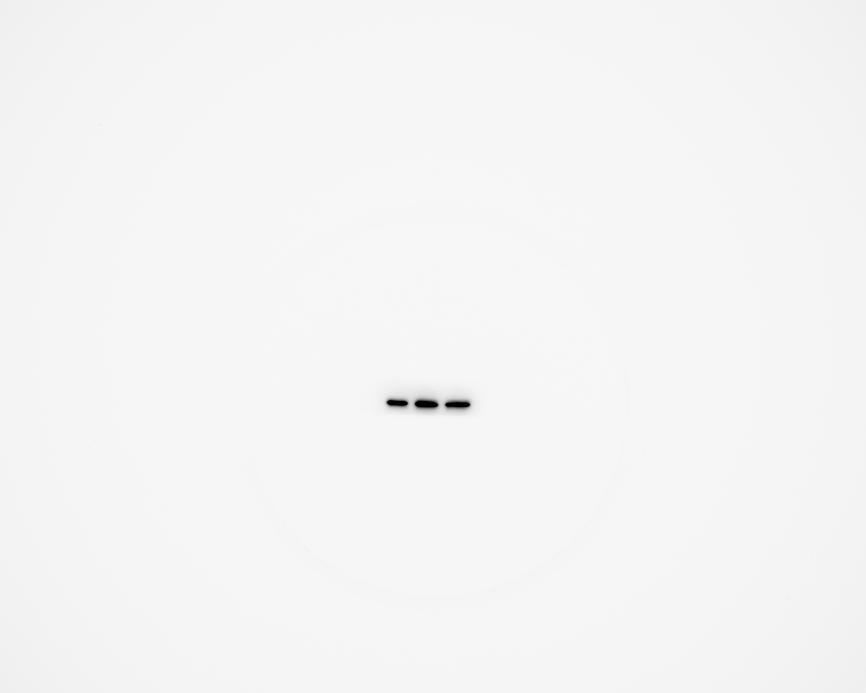
**

**U87MG CHOP**

**
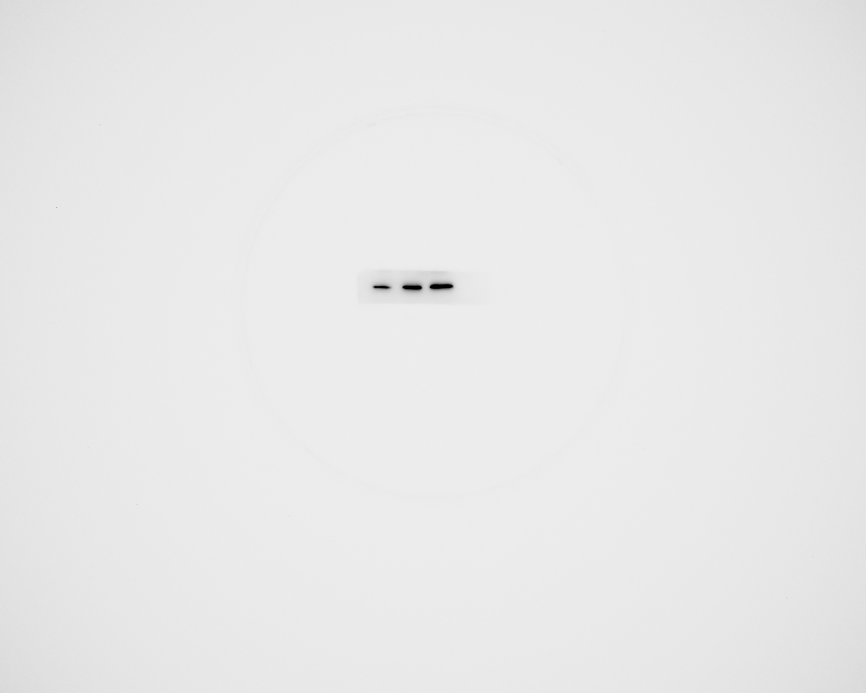
**

**U87MG eIF2α**

**
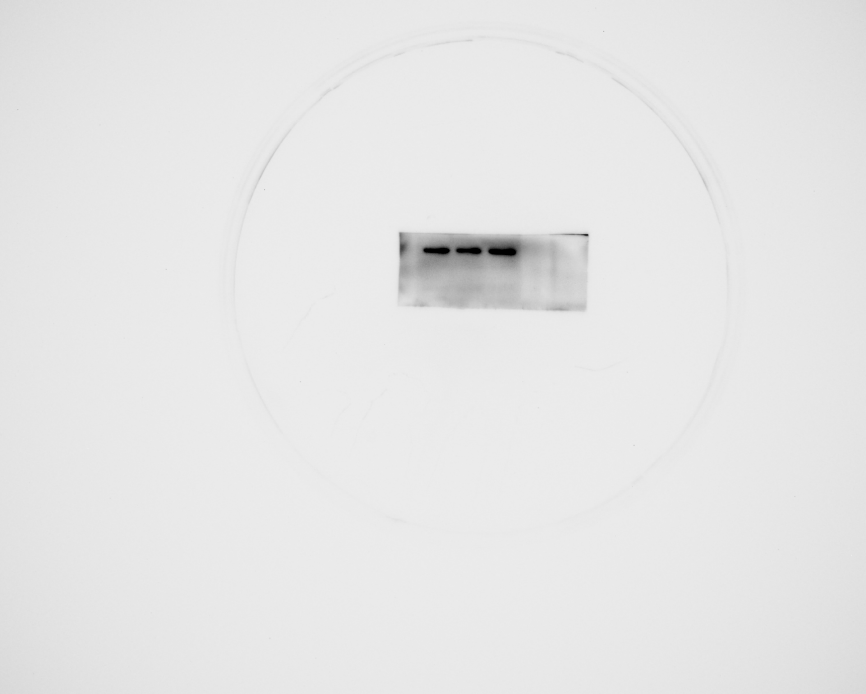
**

**U87MG p-eIF2α**

**
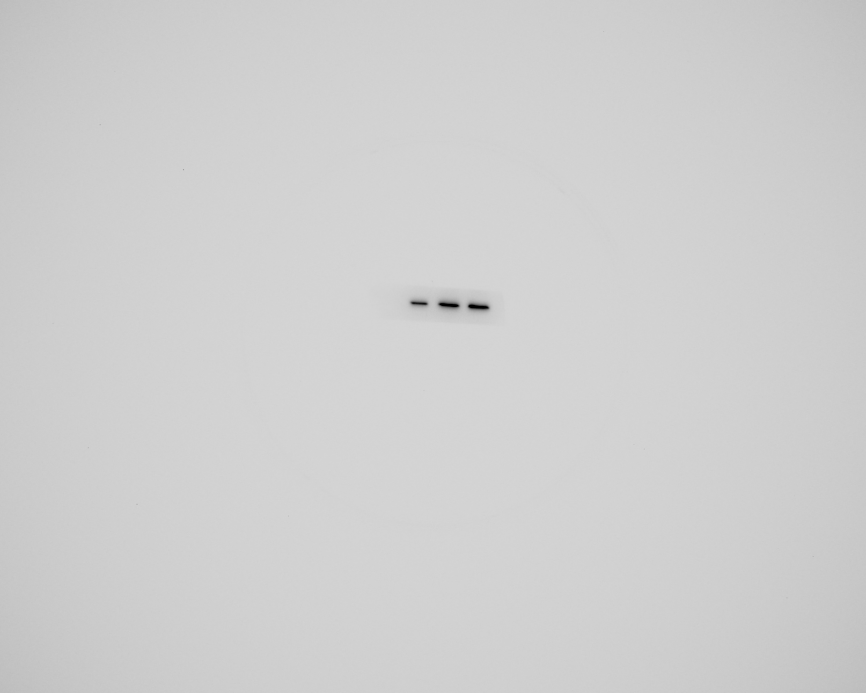
**
